# Incidence and Characteristics of Revision Adenoidectomy Among Pediatric Patients at King Abdulaziz University Hospital in Saudi Arabia

**DOI:** 10.7759/cureus.7945

**Published:** 2020-05-03

**Authors:** Shouq Alsharif, Sarah Alessa, Salwa Alshiqayhi, Ebtihaj AlAmoudi, Futoon Alobiri, Sara Amro, Hisham Alem

**Affiliations:** 1 Medicine, Kind Abdulaziz University Hospital, Jeddah, SAU; 2 Medicine, King Abdulaziz University Hospital, Jeddah, SAU; 3 Otolaryngology, King Abdulaziz University Hospital, Jeddah, SAU

**Keywords:** adenoid regrowth, tonsillectomy, pediatric patients, adenoidectomy

## Abstract

Background

Adenoidectomy is the most commonly performed pediatric operation worldwide and one of the most frequent otorhinolaryngological procedures. It is a safe procedure with a low risk of complications. However, after a successful adenoidectomy, few patients experience symptoms of nasal obstruction, suggestive of adenoid regrowth. Because of various risk factors, patients require a revision adenoidectomy. This study aimed to determine the incidence of revision adenoidectomy at King Abdulaziz University Hospital (KAUH). Moreover, we aimed to identify the characteristics and factors that present a risk of revision adenoidectomy in pediatric patients.

Materials and Methods

We retrospectively reviewed the medical records of 680 pediatric patients (age below 18 years) of Saudi and non-Saudi descent who underwent a prior adenoidectomy with or without tonsillectomy, as well as those who underwent a revision adenoidectomy. The data from 2015 to 2018 were obtained from the hospital medical records using a data collection sheet.

The data were entered on to a Microsoft excel sheet, and descriptive statistical analysis was performed using IBM SPSS software V21 (IBM Corp., Armonk, NY).

Results

The incidence of revision adenoidectomy at our center was 2.79%. We found significant relationships between comorbidities and revision adenoidectomy (p=0.014), initial adenoidectomy without tonsillectomy and revision adenoidectomy (p=0.001), and a young age at initial surgery and revision adenoidectomy. The mean age at initial adenoidectomy was 2.5 years (standard deviation [SD], ﻿±0.607 years), whereas that at revision adenoidectomy was 1.89 years (SD, ±0.737 years). The mean interval between primary and revision adenoidectomies was 42.32 months (range, 9-86 months).

Conclusion

The incidence of revision adenoidectomy at KAUH was 2.79%. Moreover, only adenoidectomy without a tonsillectomy presented a high risk of adenoid regrowth necessitating a revision adenoidectomy. Therefore, we recommend counseling patients to undergo an adenoidectomy with tonsillectomy to reduce the risk of revision adenoidectomy.

## Introduction

The adenoid is an enlarged lymphatic tissue located in the upper pharynx and plays an important role in the upper respiratory tract functions and is implicated in pediatric respiratory tract infections [1]. Adenoidectomy is the most frequently performed pediatric operation globally, and among the most common otorhinolaryngological procedure [2,3]. Adenoidectomy is primarily performed to treat clinical conditions, such as recurrent otitis media, rhinosinusitis, and nasopharyngeal airway obstruction [2]. It is generally regarded as a safe procedure with a low risk of complications [4]. In addition, the postoperative outcomes are usually good. According to the literature, 70%-100% of patients who underwent adenoidectomy experience improvement of symptoms and quality of life [5-7]. However, postoperatively, few patients experience obstructive nasal symptoms suggestive of adenoid regrowth after a successful adenoidectomy. A revision adenoidectomy may be considered in these patients after excluding other pathologies such as nasal allergies, septal deviation, and turbinate hypertrophy [7]. Risk factors that have been identified from the literature include a young age, gastroesophageal reflux disease (GERD), and a primary adenoidectomy without tonsillectomy [8,9]. A 2017 case-control study in Saudi Arabia assessed the risk factors for postoperative adenoid recurrence among patients who underwent curette adenoidectomy. The study identified that children with obstructive sleep apnea, age <5 years at the time of the first adenoidectomy, those who underwent adenoidectomy without tonsillectomy, presence of comorbid allergic rhinitis or bronchial asthma, and those with a large-sized adenoid on preoperative endoscopic examination had an increased risk of adenoid recurrence [10]. Moreover, a Taiwanese cohort study conducted in 2017 reported a higher incidence of revision adenoidectomy in boys than in girls, those belonging to a younger age group and in those who previously underwent a nasal or ventilation tube insertion [11]. Limited studies have previously investigated this aspect. Therefore, this study aimed to determine the incidence of revision adenoidectomy in King Abdulaziz University Hospital (KAUH) and identify the characteristics and risk factors for revision adenoidectomy in pediatric patients who previously underwent an adenoidectomy.

## Materials and methods

Ethical approval was obtained for this retrospective study from the institutional review board of KAUH. This study reviewed the medical records of 681 Saudi and non-Saudi pediatrics patients below 18 years of age, deposited with the otolaryngology department of KAUH, Jeddah, Saudi Arabia, in 2018. These patients included those who underwent an initial adenoidectomy with or without tonsillectomy, as well as those who underwent a revision adenoidectomy. All the data from 2015 to 2018 were obtained from the medical records using data collection sheet. The data sheet included the demographic data, such as name, nationality, and sex. Moreover, it had information about previous examinations, including the risk factors and characteristic of all the revision adenoidectomy cases, age at initial adenoidectomy, indication for initial and revision adenoidectomies, surgical techniques employed for adenoidectomy, and concurrent procedures if any, medical comorbidities such as asthma, chronic tonsillitis, and allergic rhinitis.The data were entered on to Microsoft excel sheet and descriptive statistical analysis was performed using IBM SPSS software V21 (IBM Corp., Armonk, NY). The frequency, chi-square analysis, and correlation measurements were performed in this study.

## Results

This study was aimed to determine the incidence of revision adenoidectomy at KAUH to identify the characteristics and risk factors of revision adenoidectomy in pediatric patients. During the period from 2015 to 2018, 680 adenoidectomies, with and without tonsillectomy, were performed in pediatric patients. Among them, 19 patients underwent a revision adenoidectomy. The incidence of revision adenoidectomy at KAUH was 2.79%. All adenoidectomies in the center are performed by curettage. Of the 19 patients examined, 11 (57.9%) were boys and 8 (42.1%) were girls. The mean age at initial adenoidectomy was 2.5 years (standard deviation [SD], ﻿±0.607 years), and the mean age at revision adenoidectomy was 1.89 years (SD, ﻿±0.737 years) (Table 1).

**Table 1:** Demographic data of patients who initially underwent only an adenoidectomy and subsequently a second revision adenoidectomy

Demographic data		Initial only adenoidectomy (n=661)	Revision adenoidectomy (n=19)
Nationality	Saudi	521 (78.8%)	8 (42.1%)
	Non-Saudi	140 (21.2%)	11 (57.9%)
Sex	Female	270 (40.8%)	8 (42.1%)
	Male	391 (59.2%)	11 (57.9%)
Mean age (years)		2.5﻿±0.607	1.89±0.737

**Figure 1:**
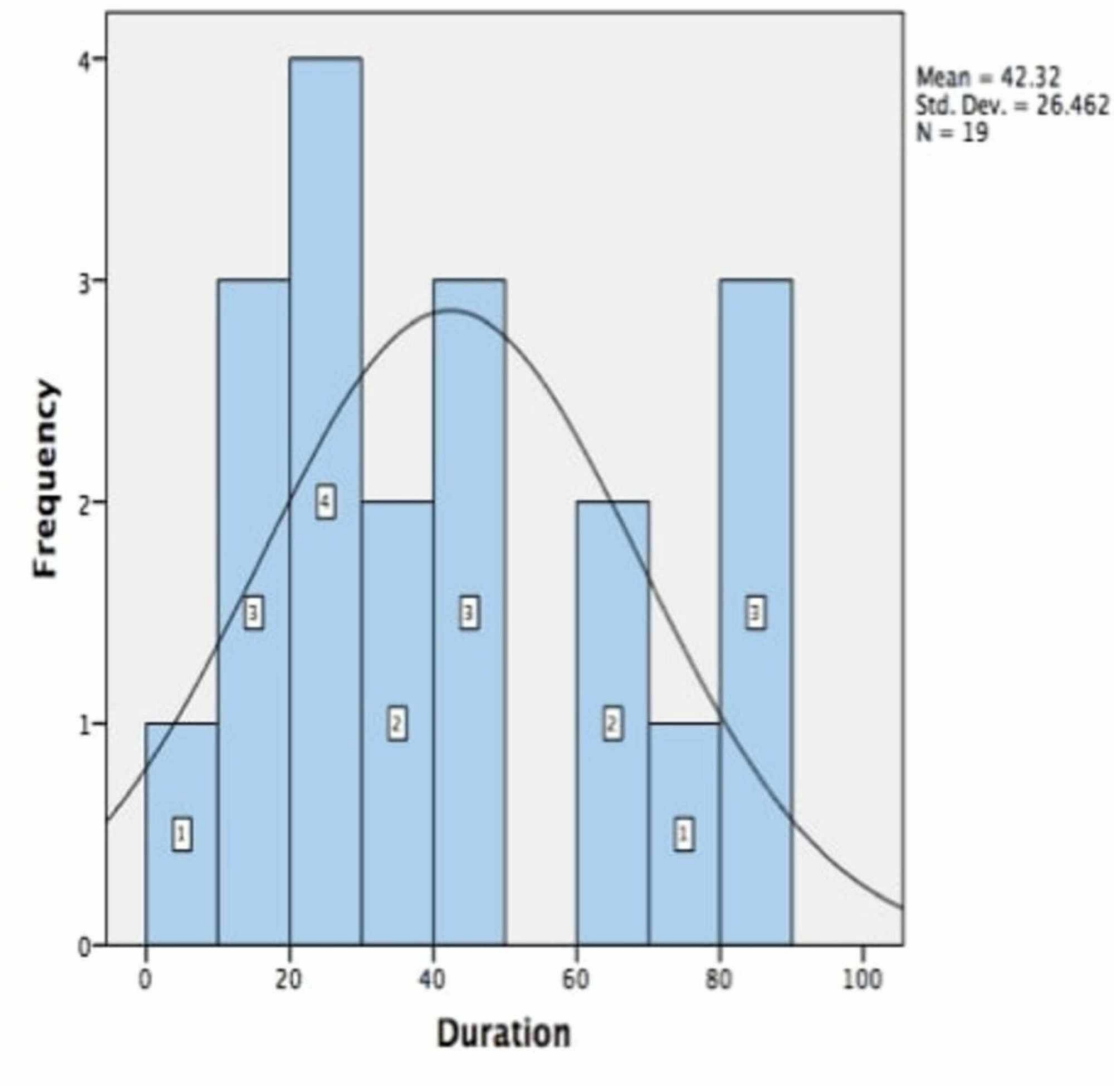
Time-frequency histogram

The mean interval between the primary and revision adenoidectomies was 42.32 months (range, 9-86 months). Figure 1 illustrates the time-frequency histogram.

Overall, 89.5% (n=17) of all patients who underwent the initial adenoidectomy had chronic adenoiditis, whereas 73.7% (n=14) patients who needed revision adenoidectomy had adenoid hypertrophy. However, according to the results of chi-square analysis, the indication for the primary surgery and that for revision surgery showed no association. A total of 31.6% patients had no comorbidities at the time of the primary adenoidectomy, whereas 47.4% patients who underwent the revision surgery had chronic tonsillitis. The analysis revealed a significant relationship between comorbidities and revision adenoidectomy (p=0.014). Table 2 shows the comorbidities at initial and revision surgeries. 

**Table 2:** Comorbidities at initial and revision adenoidectomies The analysis revealed a significant relationship between comorbidities and revision adenoidectomy (p=0.014).

Comorbidities	Frequency of patients who underwent initial surgery	Percentage of patients who underwent initial surgery	Frequency of patients who underwent revision surgery	Percentage of patients who underwent revision surgery
Chronic tonsillitis	1	5.3%	9	47.4%
Otitis media	4	21.1%	3	15.8%
Allergic rhinitis	1	5.3%	0	0
Tonsil hypertrophy	4	21.1%	0	0
Chronic tonsillitis, otitis media	1	5.3%	0	0
Chronic tonsillitis, allergic rhinitis	1	5.3%	1	5.3%
Allergic rhinitis, otitis media	1	5.3%	2	10.5%
None	6	31.6%	4	21.1%
Total	19	100%	17	100%

The percentage of tonsillectomies performed with revision adenoidectomy was higher compared with that during the initial adenoidectomy.

**Figure 2:**
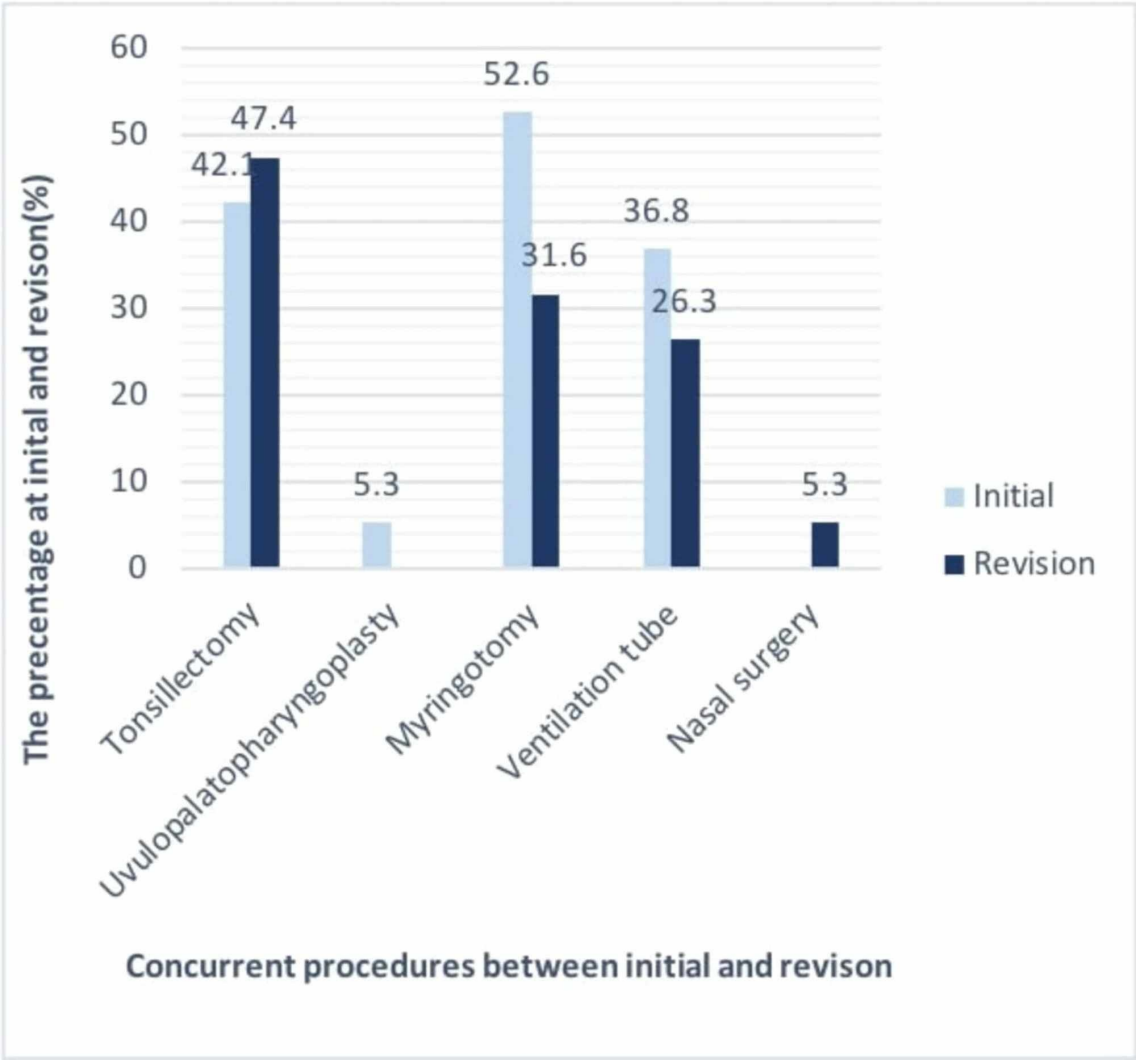
Percentages of all concurrent procedures performed with initial and revision adenoidectomies

Figure 2 illustrates the percentages of all concurrent procedures performed with the initial and revision adenoidectomies.

**Figure 3:**
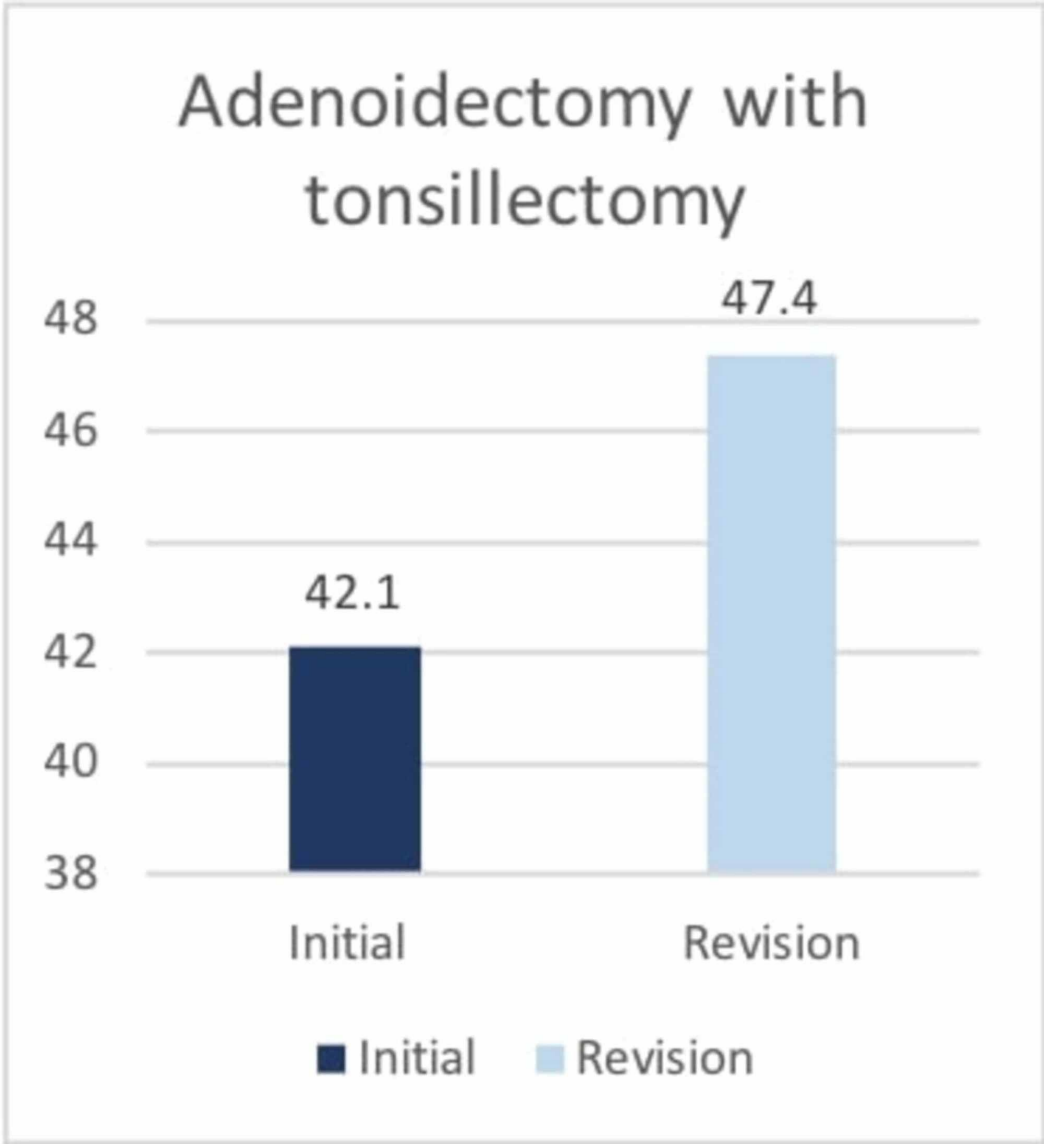
Percentages of pediatric patients who underwent only adenoidectomy and those who underwent adenoidectomy with tonsillectomy

Moreover, the results showed a significant relationship between pediatric patients who underwent initial adenoidectomy without tonsillectomy and those who underwent a second adenoidectomy (p=0.001). Figure 3 illustrates the percentages of patients who underwent adenoidectomy as well as adenoidectomy with tonsillectomy. 

## Discussion

This study determined the incidence of revision adenoidectomy cases at KAUH, and moreover, identified the patient characteristics and risk factors for a revision adenoidectomy in pediatric patients. 

Adenoidectomy is the most commonly performed procedure in the pediatric age group [2]. It is considered to be a safe procedure with low rates of complications [5]. However, the otolaryngologists are concerned with the possibility of postoperative adenoid regrowth which warrants a revision adenoidectomy. The incidence of revision adenoidectomy in our study was 2.79%, which is similar to that in previously published studies (2.7% and 2.5 %) [2,10].

In this study, 19 patients (57.9%) who underwent a revision adenoidectomy were boys, slightly higher as compared with girls. However, a previous study from Taiwan has reported similar results [4]. Nevertheless, our analysis showed no significant difference between patient sex and incidence of revision surgery.

According to the studies by the Canadian Case-Control of Repeated Adenoidectomy in Children and the American retrospective of Factors Associated with Revision Adenoidectomy, more than 2.5% children below the age of five years needed revision adenoidectomy [8,9]. In addition, a previous case-control study conducted in Saudi Arabia reported that children who are below the age of five years and underwent a primary adenoidectomy are twice as likely to require a second revision surgery [10].

In the present study, 47.4% (n=9/19) patients were between the ages of two and four years while undergoing the initial adenoidectomy. These results show the association between a young age at primary surgery and the risk of revision adenoidectomy. According to the study by Dearking et al., this significant relationship can be attributable to the small size of the nasopharynx in pediatric patients because of which complete excision of the adenoid gland might not be feasible to ensure the safety of the adjacent structures. Furthermore, the strong immune responses elicited by pediatric patients can induce hypertrophy of the residual adenoid tissue [8].

According to a retrospective study conducted in Germany in 2013, the most common indications for revision adenoidectomy were recurrent of upper airway infections or chronic otitis media with effusion [12].

However, in the present study, adenoid hypertrophy was the main indication for both the initial and revision adenoidectomies. The study results revealed no significant relationship between the indication for surgery and revision adenoidectomy. Additionally, various comorbidities play important roles in the enlargement of the adenoid, such as otitis media and gastroesophageal reflux; they have been associated with the risk of revision adenoidectomy [2]. In the present study, chronic tonsillitis was the most frequent comorbidity in pediatric patients who underwent revision surgery. Patients who were diagnosed with chronic tonsillitis might additionally have GERD. Chronic tonsillitis is a subtype of pharyngitis and is primarily caused by infection and GERD [4]. However, this study did not investigate the role of GERD because of the unavailability of data in the reviewed medical records. Therefore, it is difficult to confirm the existence of GERD as a potential comorbidity in the patients who underwent a revision adenoidectomy. However, an American study on the incidence of acid reflux in young children who underwent adenoidectomy reported the diagnosis of GERD before or after adenoidectomy and stated that it is associated with revision adenoidectomy. GERD causes respiratory mucosal inflammation and edema [8,13]. A 2013 American study identified the association of the oropharynx and middle ear with the nasopharynx, which is the anatomical location of the adenoid. Therefore, inflammation at these sites could potentially induce tissue hypertrophy [14].

Surgeons do not regularly perform tonsillectomy during initial adenoidectomy. However, two case-control studies reported that children who underwent adenoidectomy without tonsillectomy have 2.5 times and 4.0 times higher risk of revision adenoidectomy than those who underwent primary adenoidectomy with tonsillectomy [9,10]. The present study along with those reported previously identifies a strong connection between adenoidectomy without tonsillectomy and the risk of revision adenoidectomy.

The main study limitation is the lack of complete clinical information in the medical records. 

## Conclusions

This study determined the incidence of revision adenoidectomy in KAUH and identified the characteristics and risk of revision adenoidectomy in pediatric patients who underwent an initial adenoidectomy.

The incidence of revision adenoidectomy at KAUH was 2.79%. Adenoidectomy without concomitant tonsillectomy might induce a high risk of revision adenoidectomy. Recommendations to the future researchers performing tonsillectomy in pediatric patients indicated for adenoidectomy to reduce the risk of revision adenoidectomy.
